# From Solid Dispersions to Enzyme-Responsive Nanocarriers: Whey Protein Isolate Nanoparticles for Enhanced Curcumin Encapsulation and Targeted Delivery

**DOI:** 10.3390/pharmaceutics18050556

**Published:** 2026-04-30

**Authors:** Marwa Megahed, Jaina Patel, Mohammad Najlah, Hachemi Kadri, Mouhamad Khoder

**Affiliations:** 1 Health, Education and Society, Knowledge Exchange and Research Institute (HES KERI) and School of Life Sciences, Pharmacy and Chemistry, Kingston University, Penrhyn Road, Kingston upon Thames, Surrey KT1 2EE, UK; k1200928@kingston.ac.uk; 2 Codis UK, 12 Rookwood Way, Haverhill, Suffolk CB9 8PB, UK; jpatel@codis.com; 3 Pharmaceutical Research Group, School of Allied Health, Faculty of Health, Education, Medicine and Social Care, Anglia Ruskin University, Chelmsford CM1 1SQ, UK; mohammad.najlah@aru.ac.uk

**Keywords:** curcumin, whey protein isolate, solid dispersion, nanoparticles, enzyme-responsive drug delivery, solubility enhancement

## Abstract

**Background/Objectives:** Curcumin (CUR) is a potent anticancer agent whose clinical application is hindered by its extremely poor aqueous solubility. This study reports the development of enzyme-responsive whey protein isolate (WPI) nanoparticles for CUR targeted delivery. **Methods:** To overcome the initial solubility barrier, CUR was first formulated as a solid dispersion with WPI using freeze-drying. This process resulted in a significant enhancement in aqueous solubility (up to 1478-fold), with CUR existing in molecular dispersion or in an amorphous state within the protein matrix as confirmed by Differential Scanning Calorimetry (DSC) and Fourier-transform infrared (FT-IR) spectroscopy. The solubilized CUR-WPI solid dispersion was subsequently used to generate nanoparticles via a thermal gelation method, avoiding the use of organic solvents or toxic chemical crosslinkers. **Results:** The resulting nanoparticles exhibited a high drug loading efficiency of 85%. In vitro release studies demonstrated minimal CUR release in physiological buffer (pH 7.4) over 24 h, whereas exposure to trypsin, a nonspecific serine protease used as an in vitro model for tumor-associated proteolytic activity, triggered rapid nanoparticle degradation and released 95% of CUR within 3 h. **Conclusions:** These findings suggest that WPI-based nanoparticles developed from solid dispersions offer a promising, biocompatible platform for the solubility enhancement and protease-triggered delivery of hydrophobic anticancer drugs.

## 1. Introduction

Nanomedicine has transformed drug delivery by enabling precise modulation of pharmacokinetics and biodistribution, thereby reducing toxicity and improving therapeutic efficacy [1]. Targeted drug release from nanoparticles is critical for maximizing therapeutic outcomes while minimizing systemic side effects, especially in cancer therapy where treatments are often highly toxic [2]. Effective nanoparticulate systems must prevent premature drug leakage during circulation while ensuring efficient release at the therapeutic site [3,4]. This typically requires a release-triggering mechanism specific to, or highly expressed within, the target environment, such as the tumor microenvironment [5].

Historically, lipid-based systems, such as liposomes, and polymeric nanoparticles have dominated the field, representing the earliest and most widely established nanomedicines in clinical use [6,7]. Protein-based polymeric nanoparticles, in particular, have attracted significant attention in targeted drug delivery research due to their biocompatibility, biodegradability, and adaptability in encapsulating a range of therapeutic agents [8]. Among natural proteins, albumin nanoparticles (Abraxane^®^) are among the few nanoparticulate systems that have successfully transitioned into clinical use for cancer treatment, receiving FDA approval in 2005 [9].

Protein nanoparticles can be prepared through various techniques, including desolvation, coacervation, self-assembly, thermal gelation, nano-spray drying, and Nab-technology [10,11]. Chemical crosslinkers, often glutaraldehyde, are widely employed to stabilize the nanoparticle matrix and improve the release profile. Despite their effectiveness, these crosslinking agents are associated with cytotoxicity, which compromises the safety profile of protein-based delivery systems. In this context, the thermal gelation method is advantageous as it induces thermal denaturation and physical crosslinking within the protein molecular structure. However, this approach offers poorer control over nanoparticle size and is unsuitable for heat-labile drugs [12]. For the encapsulation of hydrophilic drugs, the drug molecules can be loaded prior to nanoprecipitation by dissolving them in the aqueous protein solution. In the case of water-insoluble drugs, emulsification of organic solvents is often needed to facilitate drug encapsulation in the hydrophilic protein nanoparticles. However, the use of organic solvents undermines the biocompatibility benefits of protein nanoparticles.

With the increasing number of poorly water-soluble new chemical entities entering the development pipeline, it is imperative that innovative solubility enhancement approaches are optimized to enable their encapsulation into protein-based nanoparticles [13,14]. The solid dispersion technique is a well-established strategy for improving the solubility of poorly water-soluble drugs [15]. In solid dispersions, drug molecules are either molecularly dispersed in a hydrophilic solid matrix, forming a solid in solid solution, or distributed as small amorphous particles within the hydrophilic carrier [16]. Upon reconstitution in water, the rapid dissolution of the hydrophilic matrix leads to the prompt release of the drug, thereby enhancing its dissolution rate and apparent solubility. Another approach involves the formation of a soluble complex, whereby hydrophobic drug molecules bind to a hydrophilic substrate and form a water-soluble complex. Khoder et al. reported the successful use of bovine serum albumin to improve the aqueous solubility of both ionizable and non-ionizable hydrophobic drugs [17,18]. In addition to dispersing drug molecules within a water-soluble protein matrix, the albumin molecules were found to form soluble complexes with drug molecules through salt bridges with ionizable compounds or hydrophobic interactions with non-ionizable ones. These interactions led to very significant enhancements in drug solubility and dissolution rate. Other macromolecular carriers that have shown potential to improve the solubility of drugs through complexation include whey proteins isolated (WPI) from milk [19].

The formation of a drug–protein solid dispersion prior to nanoprecipitation is advantageous for two main reasons. First, reconstitution of the drug–protein solid dispersion in water significantly enhances drug solubility, thereby improving the encapsulation efficiency of hydrophobic drugs within protein nanoparticles. Second, in the context of cancer-targeted therapy, the solid dispersion formation can chemically “lock” drug molecules within the protein matrix, effectively hindering premature drug release prior to reaching the therapeutic target (i.e., during systemic circulation). Cancerous tissues are known to overexpress proteolytic enzymes relative to healthy tissues, a biochemical distinction that provides a valuable opportunity for site-specific drug release at the tumor microenvironment [20]. For instance, biodegradable microgels have been engineered to achieve triggered release in response to specific enzymes like lysozyme, demonstrating the precision of such an approach [21].

Curcumin (CUR) is a hydrophobic polyphenol widely recognized for its therapeutic potential, particularly its potent anticancer activity [22,23,24]. However, its clinical application is severely limited by poor aqueous solubility (reported to be in the range of 0.011–0.6 mg/L) and consequently low bioavailability [25]. This study presents a novel approach that integrates solid dispersion technology with enzyme-responsive (WPI) nanoparticles to enhance CUR solubility and enable protease-triggered drug release. This dual-stage strategy first utilizes freeze-dried CUR–WPI solid dispersions to achieve molecular drug dispersion. Subsequently, WPI nanoparticles are fabricated via thermal gelation without organic solvents or chemical crosslinkers. The objective of this work was to evaluate CUR solubility enhancement and characterize the resulting nanoparticles. Furthermore, protease-triggered release was investigated in the presence of trypsin to establish the potential of this platform for targeted drug delivery.

## 2. Materials and Methods

### 2.1. Materials

CUR and Whey Isolate™ were purchased from Acros Organics (Geel, Belgium) and Bulk Powders^®^ (Colchester, UK), respectively. Trypsin, hydrochloric acid (HCl), and ethanol were obtained from Sigma-Aldrich (Gillingham, UK). Millex-GS sterile syringe filters (pore size 0.22 µm, 33 mm) were obtained from Millipore, Merck KGaA (Darmstadt, Germany).

### 2.2. Solid Dispersion Preparation and Characterization

#### 2.2.1. Solid Dispersion Preparation

CUR (0.3 g) was dissolved in 5 mL ethanol and gradually added to 95 mL of WPI solution (1.75% *w*/*v*). The resulting suspension (10:1 CUR:WPI molar ratio) was mixed for 20 min, frozen with liquid nitrogen and subsequently freeze-dried for 48 h at 0.1 mbar with a condenser temperature of −55 °C (VirTis BenchTop Pro, SP Scientific, Ipswich, UK). The sample was labeled as CUR:WPI-SD-1 and stored in the fridge.

To obtain a fully soluble solid dispersion (labeled CUR: WPI-SD-2), a second batch was prepared as outlined above. Once removed from the freeze dryer, the solid dispersion was added to 100 mL of water and placed in a shaking water bath for 24 h at 25 °C, before being filtered through Millipore filters (0.22 µm), frozen in liquid nitrogen, and freeze-dried for a second time.

#### 2.2.2. Fourier-Transform Infrared Microscopy (FT-IR)

Fourier-transform infrared (FT-IR) spectra of CUR, WPI, and CUR:WPI-SD-1 were recorded using an FT-IR spectrometer (Thermo Scientific Nicolet iS5, Thermo Fisher, Madison, WI, USA). Spectra were acquired over the wavenumber range of 4000–500 cm^−1^ at ambient temperature.

#### 2.2.3. Differential Scanning Calorimetry (DSC)

DSC analysis of WPI, CUR, WPI:CUR-SD-1 and WPI:CUR-SD-2 was performed using differential scanning calorimeter (Mettler Toledo DSC822e, Mettler-Toledo Limited, Leicester, UK). Each sample (1–3 mg) was weighed and crimp-sealed in an aluminum pan before piercing the lid to maintain constant pressure. The pans were loaded into the sample holder and thermograms were obtained under nitrogen gas flow (20 mL/min) across 25–400 °C at a heating rate of 10 °C/min. Analysis of thermograms was performed using STAReSW 10.00 software.

#### 2.2.4. Aqueous Solubility and Dissolution Studies

The shake-flask method was used to determine the aqueous solubility of CUR, CUR physically mixed with WPI (CUR:WPI-PM), and the solid dispersion (WPI:CUR-SD-1). For CUR:WPI-PM, an excess amount of CUR (10 mg) was physically mixed with increasing amounts of WPI in the solid state, and each mixture was then added to 3 mL of water to obtain final WPI concentrations of (0 (CUR only, no WPI), 0.3, 0.9, 1.8 and 3.6 mM). For WPI:CUR-SD-1, increasing amounts of solid dispersion were reconstituted in vials containing 3 mL of water to yield equivalent (0.3, 0.9, 1.8, and 3.6 mM) WPI concentrations. The vials were placed in a shaking water bath at 25 °C for 24 h to reach equilibrium. The samples were then filtered through 0.22 µm hydrophilic membrane filters (MF-Millipore™, Merck Group, Darmstadt, Germany) and UV absorbance of the filtrate was measured at 425 nm using a UV spectrometer (JENWAY, Dunmow, Essex, UK). A standard calibration curve CUR was created in ethanol (R^2^ ≥ 0.999) and used to calculate the CUR solubility in water.

In vitro dissolution studies were performed to evaluate the dissolution behavior of CUR from different formulations. Free CUR (10 mg), CUR:WPI-SD-1 and CUR:WPI-SD-2 (182.5 mg) were conducted in 50 mL of water at 25 °C under constant stirring (100 rpm). A dissolution volume of 50 mL was selected to maintain sink conditions, while allowing the detection of early release concentrations. At predetermined time intervals (0, 5, 10, 15, 20, 30, 45, and 60 min), 2 mL aliquots were withdrawn and immediately replaced with an equal volume of fresh medium. The absorbance was measured directly with a UV spectrophotometer blanked with 0.365% WPI solution and the CUR concentrations and percentage release were calculated using the calibration curve mentioned above.

### 2.3. Preparation and Characterization of CUR-Loaded WPI Nanoparticles

#### 2.3.1. Nanoparticle Preparation

CUR:WPI-SD-2 (150 mg) was dissolved in 50 mL deionized water to make a 0.3% *w/v* solution. The pH was adjusted to 5.8 by adding HCl (0.01 M) dropwise. The solution was then immersed into a shaking water bath at 85 °C for 15 min to facilitate the unfolding and aggregation of WPI. The resulting nanodispersion was then removed from the water bath and left to cool to room temperature. One milliliter of the sample was retained to measure the particle size and surface charge using the Zetasizer Nano series (Malvern instruments Ltd., Malvern, UK). The remaining solution was centrifuged at 25,000 rpm for 30 min using aSigma 3–30 KS ultracentrifuge, (Sigma Laborzentrifugen GmbH, Osterode am Harz, Germany) and the pellet was freeze-dried for 48 h.

#### 2.3.2. Scanning Electron Microscope (SEM)

The freeze-dried CUR-loaded WPI nanoparticles were bound to 12 mm aluminum stubs with the use of carbon sticky tabs and observed under the microscope (Zeiss Evo50, Oxford Instruments, Abingdon, UK) operated at an accelerating voltage of 30 kV under low-vacuum mode.

#### 2.3.3. Particle Size and Surface Charge Analysis

The nanoparticles particle average size (hydrodynamic diameter), polydispersity index (PDI), and ζ-potential were determined by the dynamic light scattering technique using the Zetasizer Nano ZS (Malvern, UK). Size analysis was carried out at an angle of 90° and at a temperature of 25 °C.

#### 2.3.4. Loading Capacity and Release Study

The release of CUR was investigated in phosphate buffer (pH 7.4) with or without 0.2% *w/v* trypsin. Briefly, 1 mL of release medium was added to Eppendorf tubes and 4 mg of nanoparticles were evenly dispersed in each of the tubes and placed in a water bath operated at 37 °C and 100 rpm. The tubes were removed from the water bath at specific time intervals (0, 15, 30, 60, 120, 180, 240 min, and 24 h) and centrifuged at 14,000 rpm and the supernatant was filtered using a 0.22 µm Millex-GS syringe filter, diluted 5-fold with ethanol, and filtered again. CUR concentration was quantified using UV-vis spectroscopy at 425 nm. As shown in the UV-vis spectra (Figure S1, Supplementary Data), the maximum absorbance wavelength of CUR is significantly resolved from the primary absorbance peak of WPI. To account for non-linear baselines and potential solvent or protein interference, all subsequent measurements were blanked using drug-free WPI solutions of corresponding concentrations.

The loading efficiency (LE, %) was calculated using the cumulative drug release at 24 h in trypsin-containing PBS buffer according to the following equation:

$$Loading\;efficiency\left(\%\right)=\frac{Amount\;of\;drug\;entrapped}{total\;Amount\;in\;CUR:WPI\text{-}SD\text{-}2}$$

The total amount in CUR:WPI-SD-2 was determined both theoretically (using the phase solubility diagram equation) and experimentally by determining the concentration of CUR in the CUR-WPI solution prior to the nanoprecipitation step.

In parallel with the release study, changes in nanoparticle size were monitored under the same incubation conditions in PBS with and without trypsin. Particle size measurements were performed using a Zetasizer, as described in Section 2.3.3.

### 2.4. Statistical Analysis

All experiments were carried out in triplicate, and the results were expressed as the mean ± standard deviation. Statistical analysis was performed using one-way analysis of variance (ANOVA) followed by Tukey’s post hoc test for multiple comparisons between groups. Statistical significance was considered when *p* < 0.05.

## 3. Results and Discussion

### 3.1. Solid Dispersion Preparation and Characterization

**Figure 1:**
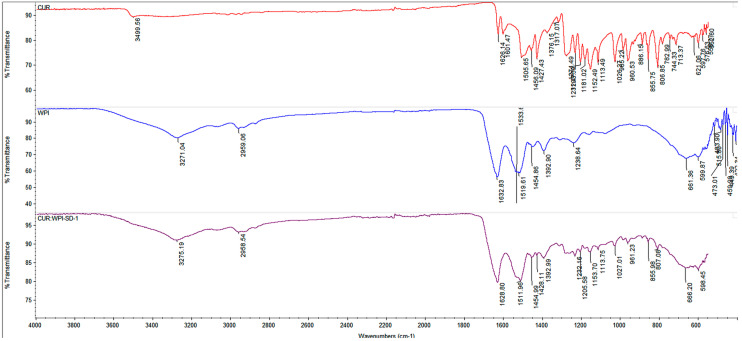
FT-IR spectra of CUR, WPI and CUR: WPI-SD-1.

The CUR:WPI-SD-1 formulation appeared as a fine orange granular powder. In contrast, the CUR:WPI-SD-2 formulation was pale yellow in color and exhibited a light and porous nature (Figure S2, Supplementary Data). The FT-IR spectrum of CUR exhibited characteristic absorption bands, including a broad O–H stretching vibration (~3499.56 cm^−1^), C=O and aromatic C=C stretching (~1626, 1601, and ~1504 cm^−1^, respectively), and a C–OH bending vibration (~1427 cm^−1^) (Figure 1). The spectrum of WPI showed typical protein features, with a broad O–H/N–H stretching band (~3271 cm^−1^), aliphatic C–H stretching (~2960 cm^−1^), and prominent amide I and amide II bands (~1632 and ~1519 cm^−1^, respectively), corresponding to C=O stretching of peptide bonds and N–H bending coupled with C–N stretching. Following CUR incorporation, the characteristic O–H stretching vibration of CUR at ~3499 cm^−1^ disappeared, and the amide I and amide II bands shifted from 1632 to ~1628 cm^−1^ and from 1519 to ~1512 cm^−1^, indicating alterations in the hydrogen-bonding environment and protein secondary structure. These spectral changes suggest the formation of non-covalent interactions, primarily hydrogen bonding and hydrophobic associations, confirming successful molecular encapsulation of CUR within the WPI matrix rather than simple physical mixing.

**Figure 2:**
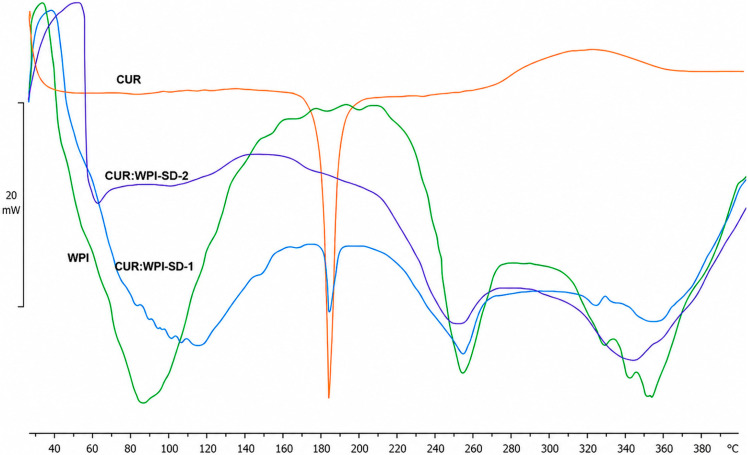
DSC thermograms of CUR, WPI, WPI:CUR-SD 1 and WPI:CUR-SD 2.

Figure 2 presents the DSC thermograms of CUR, WPI, WPI:CUR-SD-1 and WPI:CUR-SD-2. CUR exhibits a sharp endothermic peak at 183 °C, corresponding to its melting point, which is also observed in WPI:CUR-SD-1, indicating CUR presence in its crystalline form. A broad endothermic peak between 75 °C and 110 °C likely corresponds to when moisture evaporation was observed. The WPI thermogram shows a broad endothermic peak (40–110 °C) linked to heat-induced unfolding of α-lactoglobulin and β-lactoglobulin, while the peaks at 250 °C and 320 °C suggest thermal degradation. In contrast, WPI:CUR-SD-2 lacks the CUR crystalline peak, indicating the complete removal of crystalline CUR form WPI dispersion and/or its conversion to amorphous form. The endothermic peak at 60 °C supports further the presence of amorphous CUR in WPI:CUR-SD-2. The conversion of drugs to the amorphous form offers advantages in terms of solubility and dissolution due to higher Gibbs free energy.

### 3.2. Solubility and Dissolution Rate Studies

**Figure 3:**
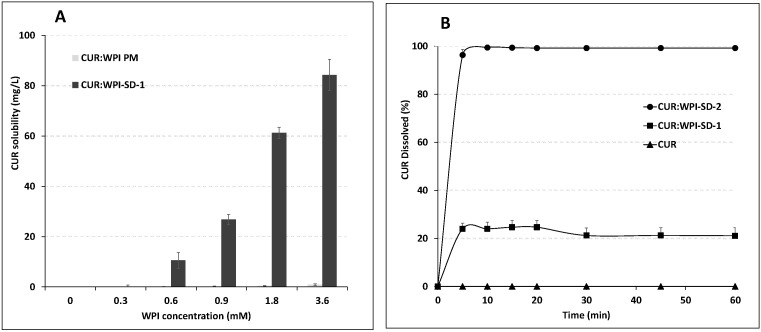
(**A**) Aqueous solubility enhancement of CUR:WPI-SD-1 compared with CUR:WPI-PM (physical mixture). Note: CUR:WPI PM solubility values are near the baseline due to the drug’s poor solubility. (**B**) In vitro dissolution profiles of CUR, CUR:WPI-SD-1 and CUR:WPI-SD-2 in water.

In line with its hydrophobic and crystalline nature, the intrinsic aqueous solubility of pure CUR was 0.057 mg/L (Figure 3A), which agrees with the literature reports that CUR is practically insoluble in water (<8 μg mL^−1^) [26]. The addition of increased concentrations of WPI to water (CUR:WPI-PM) led to significant enhancement (*p* < 0.05) in CUR solubility for the physical mixture, reaching approximately 0.91 mg/L at 3.6 mM WPI, corresponding to an enhancement factor of ~16 relative to pure CUR (Figure 3A). A substantially greater increase in CUR solubility was observed for the CUR:WPI-SD-1 formulation, reaching 84.3 mg/L (at 3.6 mM of WPI) with an enhancement factor of 1478 compared to pure CUR. This pronounced increase highlights the effectiveness of the solid dispersion approach in improving CUR solubilization.

**Figure 4:**
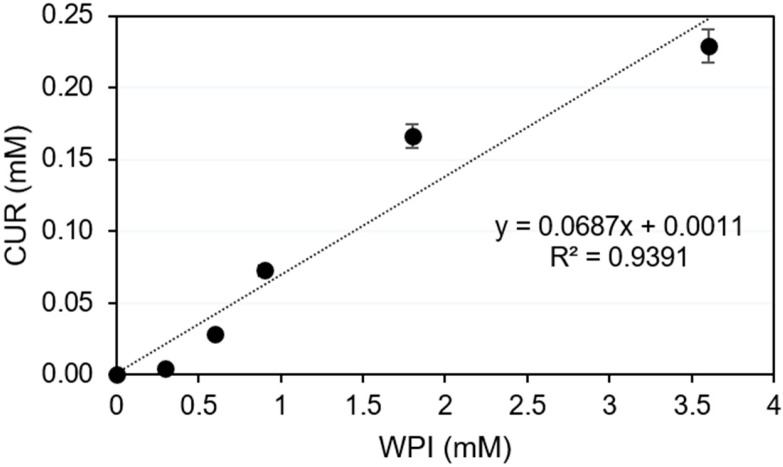
Phase solubility diagram of CUR:WPI-SD-1.

The phase solubility diagram (Figure 4) shows a linear relationship with a slope of 0.0685, suggesting a CUR:WPI complexing ratio of around 0.07 (i.e., around 15 molecules of WPI were needed to dissolve one CUR molecule). Visually, the enhancement of CUR solubility was associated with increased solution color intensity as WPI concentration increases (Figure S3, Supplementary Data). WPI consists of several proteins, including mainly lactoglobulins, bovine serum albumin and immunoglobulin. The β-lactoglobulin and bovine serum albumin fractions in WPI have a high affinity for hydrophobic compounds such as CUR [17,19]. The fact that almost 15 WPI molecules were required to solubilize one CUR molecule suggests that only a small portion of WPI is made up of proteins (potentially the albumin fraction representing 10% of WPI mass) with the ability to form strong binding complexes [17,18].

Figure 3B shows the dissolution profiles of CUR, CUR:WPI-SD-1 and CUR:WPI-SD-2 in water. Free CUR showed no dissolution due to a lack of aqueous solubility. Dissolution profiles of both solid dispersions showed an immediate release which could be attributed to increased solubilization and improved wettability of CUR in WPI matrix. While 97% of CUR in CUR:WPI-SD-2 dissolved in the first 5 min, a maximum of 20% of CUR was released from CUR:WPI-SD-1 in one hour. The partial crystallinity of CUR observed in the DSC thermogram of CUR:WPI-SD-1 suggests that a fraction of the drug remained undissolved in the aqueous medium. On the other hand, 100% of drug from CUR:WPI-SD 2 was released since CUR is homogenously dispersed in the WPI in the amorphous form, confirmed by DSC analysis. A similar observation was reported previously by Khoder et al. in bovine serum albumin-based solid dispersion [18].

### 3.3. Nanoparticle Preparation and Characterization

**Figure 5:**
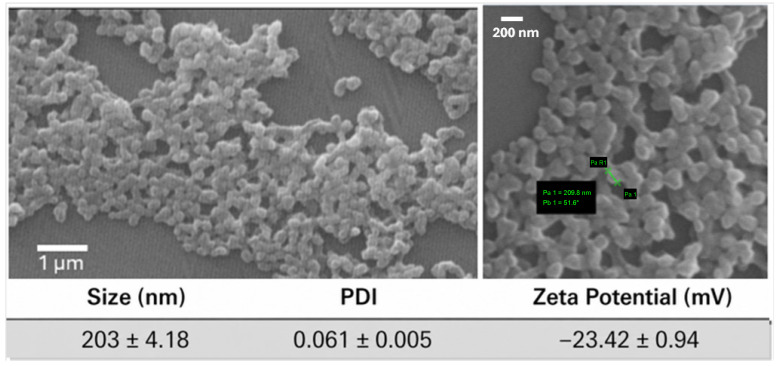
Scanning electron microscopy (SEM) images of CUR-loaded WPI nanoparticles at different magnifications (scale bars: 1 µm, **right** image; 200 nm, **left** image). The accompanying table summarizes the average particle size, polydispersity index (PDI), and ζ-potential of the nanoparticles.

CUR-loaded WPI nanoparticles were prepared using a reconstituted CUR:WPI-SD-2 solution (0.3% *w*/*v*—corresponding to approximately 0.18 mM WPI). The complete and rapid dissolution of CUR achieved in CUR:WPI-SD-2 represents a key advantage of this approach, since it ensures that CUR is available in a fully solubilized molecular state prior to nanoparticle formation. This eliminates the presence of undissolved crystalline drug, which can otherwise limit encapsulation efficiency and reproducibility. Furthermore, undissolved drug may precipitate during centrifugation within the nanoparticle pellet, leading to an overestimation of drug loading. The thermal gelation method (85 °C) used in this study initiated WPI molecule unfolding, aggregation, and subsequent nanoprecipitation. With a relatively small molecular weight (approximately 17–20 kDa), WPI exhibits high unfolding capacity, enabling controlled nanoprecipitation [27] and bypassing the use of organic solvents and chemical crosslinkers. The SEM image showed that CUR-loaded WPI nanoparticles had a slightly elongated spherical shape with an estimated size ranging between 200 nm and 250 nm (Figure 5). The particle size observed under SEM was in the same order of magnitude as the measurements obtained by the Zetasizer (Figure 5). It should be noted that Zetasizer measurements represent the hydrodynamic diameter of hydrated particles in suspension, which is typically larger than the dry particle size observed by SEM. The narrow PDI indicated the uniformity of nanoparticles size while the zeta potential of WPI nanoparticles was around −23.5 mV. Prior to nanoprecipitation, the pH of CUR-WPI-SD-2 solution was adjusted at 5.8, which is just above the isoelectric point of WPI (pH 5.5). At this pH, deprotonation of carboxyl groups led to a net negative surface charge.

The drug loading efficiency (LE) was calculated using the equation of the phase solubility diagram (Figure 4), which indicates that one CUR molecule requires approximately 15 WPI molecules to achieve dissolution. Given that CUR–WPI–SD-2 is fully soluble in water (Figure 3B), the 4 mg of nanoparticles used in the release study would contain a maximum CUR mass of approximately 5.5 µg. A comparable CUR mass was obtained experimentally by determining the concentration of CUR in the CUR:WPI-SD-2 solution used for nanoprecipitation. The LE was 85%, which is relatively high for a hydrophobic molecule encapsulated within a hydrophilic matrix. This can be attributed to the complexation of CUR with WPI in CUR:WPI–SD-2, which effectively locks CUR molecules within the nanoparticles. Importantly, this same characteristic is expected to limit premature CUR release in the bloodstream, thereby enhancing the targeting potential of the nanoparticles.

**Figure 6:**
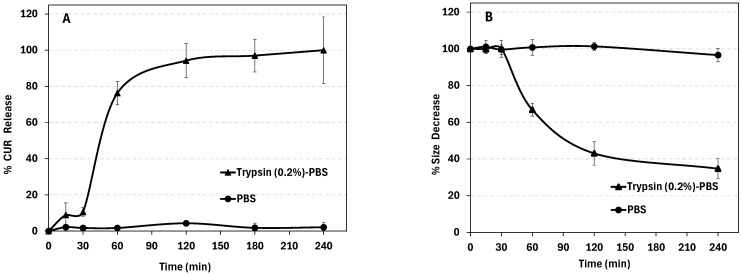
(**A**) CUR release (%) in PBS and trypsin (0.2%) containing PBS. (**B**) WPI nanoparticle size reduction (%) in PBS and trypsin (0.2%) containing PBS.

Figure 6A shows that CUR release from WPI nanoparticles is highly enzyme-responsive. In PBS, simulating the pH of blood circulation, no detectable release was observed over 4 h (and it remained negligible even after 24 h indicating good nanoparticle stability and minimal premature drug leakage. In contrast, in the presence of trypsin, used to model elevated protease activity in tumor environments, CUR release was rapid: ~10% in the first 30 min, ~60% within 1 h, and ~95% by 3 h. Correspondingly, nanoparticle size decreased by ~60% over 4 h (Figure 6B), consistent with enzymatic degradation of the WPI matrix. Comparable findings were reported by Li et al., where thermally self-assembled albumin nanoparticles exhibited more than 50% degradation within an hour in a trypsin-containing medium [28].

These results suggest a protease-triggered, degradation-controlled release: the nanoparticles remain intact in circulation-mimicking medium but rapidly degrade and release CUR in enzyme-rich tumor environments, potentially enhancing tumor targeting while minimizing systemic exposure. However, it is important to acknowledge that these in vitro conditions may not fully replicate the complexity of the tumor microenvironment, where a diverse array of enzymes and biological barriers could influence the actual release rate.

## 4. Conclusions

This study successfully demonstrates the dual functionality of WPI as both a potent solubility enhancer and a “smart” nanocarrier for hydrophobic drugs. By employing a solid dispersion technique, we achieved a 1478-fold increase in the aqueous solubility of CUR, effectively converting the crystalline drug into an amorphous state and water-soluble molecular complex. This pre-solubilization step was critical, enabling the subsequent fabrication of high-loading nanoparticles via a thermal gelation method that eliminated the need for organic solvents and toxic chemical crosslinkers. The resulting nanoparticles exhibited excellent stability in physiological conditions, effectively preventing premature drug release, which is a common limitation in conventional protein-based carriers. Crucially, the system displayed a sharp, protease-triggered release profile, with rapid cargo liberation occurring only in the presence of proteolytic enzymes. This specific responsiveness highlights the potential of these nanocarriers for stimulus-responsive drug delivery within the tumor microenvironment, where protease activity is elevated. While these in vitro results provide a strong proof-of-concept, further in vivo studies are required to validate the performance and release specificity of this system within the complex biological environment. Overall, this work establishes WPI-based solid dispersions as a versatile, biocompatible, and cost-effective platform for overcoming solubility challenges while conferring enzyme-responsive targeting capabilities to anticancer therapeutics.

## Data Availability

The original contributions presented in this study are included in the article/Supplementary Materials. Further inquiries can be directed to the corresponding authors.

## Supplementary Materials

The following supporting information can be downloaded at https://www.mdpi.com/article/10.3390/pharmaceutics18050556/s1, Figure S1: UV-Vis spectrum of WPI in water and curcumin in ethanol; Figure S2: Physical appearance of CUR:WPI-SD-1 (left) and CUR:WPI-SD-2 (right); Figure S3: CUR aqueous solutions in the presence of WPI in serial concentrations (mM). Physical mixture (left) and solid dispersion (right).
