# Supplementary material for: From Solid Dispersions to Enzyme-Responsive Nanocarriers: Whey Protein Isolate Nanoparticles for Enhanced Curcumin Encapsulation and Targeted Delivery
Source: Pharmaceutics. 2026 Apr 30;18(5):556. doi: 10.3390/pharmaceutics18050556 (PMC13210158; doi:10.3390/pharmaceutics18050556)
Supplement: Supplementary file 1 [file pharmaceutics-18-00556-s001.zip › pharmaceutics-4199644-supplementary.pdf]

Supplementary Materials

From Solid Dispersions to Enzyme-Responsive Nanocarriers: Whey Protein Isolate Nanoparticles for Enhanced Curcumin Encapsulation and Targeted Delivery

Marwa Megahed [1], Jaina Patel [1,2], Mohammad Najlah [3], Hachemi Kadri [1*], Mouhamad Khoder [1*]

1 Health, Education and Society, Knowledge Exchange and Research Institute (HES KERI) and School of Life Sciences, Pharmacy and Chemistry, Kingston University, Penrhyn Road, Kingston upon Thames, KT1 2EE, UK
2 CODIS UK, 12 Rookwood Way Haverhill CB9 8PB, UK
3 Pharmaceutical Research Group, School of Allied Health, Faculty of Health, Education, Medicine and Social Care, Anglia Ruskin University, Chelmsford, CM1 1SQ, UK

Correspondence: h.kadri@kingson.ac.uk, M.khoder@kingston.ac.uk

The supplementary materials include the following contents:

1.Figure S1………………………………………………………………………………… 1
2.Figure S2………………………………………………………………………………… 2
3.Figure S3…………………………………………………………………………………2

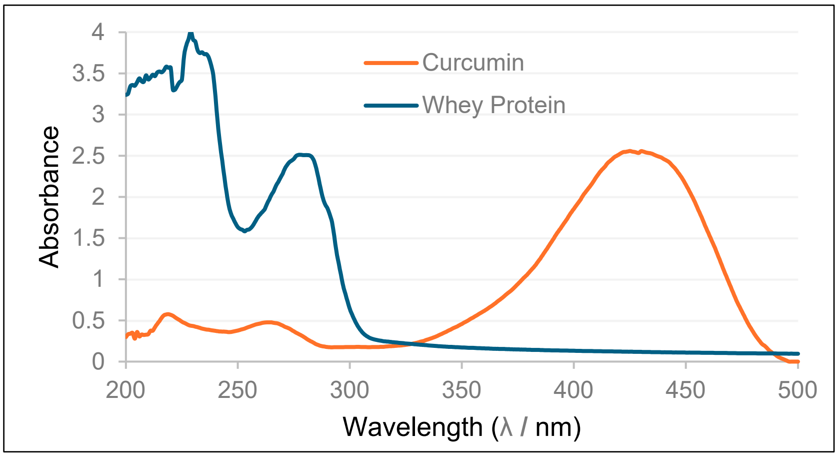

Figure S1. UV-Vis spectrum of WPI in water and curcumin in ethanol

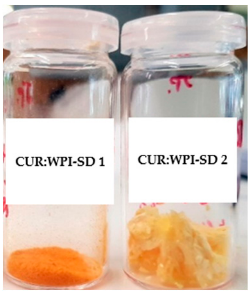

Figure S2. Physical appearance of CUR:WPI-SD-1 (left) and CUR:WPI-SD-2 (right)

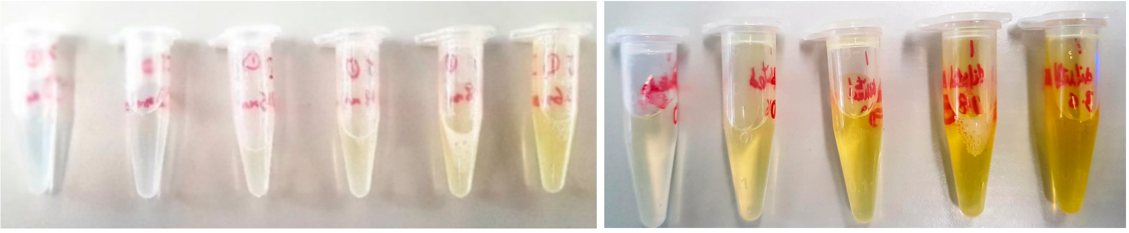

Figure S3. CUR aqueous solutions in the presence of WPI in serial concentrations (mM). Physical mixture (left) and Solid Dispersion (right).
